# Successful treatment of recurrent volvulus in the afferent limb of the pouch following proctocolectomy for ulcerative colitis: a case report

**DOI:** 10.1186/s40792-020-01007-4

**Published:** 2020-09-24

**Authors:** Michio Itabashi, Yoshiko Bamba, Hisako Aihara, Kimitaka Tani, Ryousuke Nakagawa, Kurodo Koshino, Takeshi Ohki, Shinpei Ogawa, Yuji Inoue, Masakazu Yamamoto

**Affiliations:** Department of Surgery, Institute of Gastroenterology, Tokyo Women’s Medical University, 8-1 Kawada-cho, Shinjuku-ku, Tokyo, 162-8666 Japan

**Keywords:** Volvulus, Pouch volvulus, Ulcerative colitis, Proctocolectomy, Afferent limb volvulus, Pouchpexy

## Abstract

**Background:**

Pouch volvulus after proctocolectomy for ulcerative colitis is a very rare postoperative complication. The common site of pouch volvulus has been reported to be the ileal pouch–anal anastomosis and the middle part of the pouch, but no reports on pouch volvulus in the afferent limb of the pouch have been observed. Here, we report the case of a patient with afferent limb volvulus who underwent afferent limbpexy, but required reoperation 7 months later.

**Case presentation:**

A 38-year-old man with refractory ulcerative colitis had undergone open proctocolectomy 10 years ago at another hospital. He had been aware of lower abdominal pain and bowel movement difficulty for 2 years. After repeated bowel obstruction, he was referred to our hospital for surgery. Based on the radiographic findings, we diagnosed a pouch volvulus and performed an operation. Laparoscopically, counterclockwise rotation of the afferent limb of the pouch was recognized. Moreover, the ileal mesentery was adhered and fixed to the presacral space 20 cm from the oral side of the pouch. The antimesenteric side of the afferent limb was fixed using interrupted stiches on the left peritoneal wall of the pelvis. He was discharged uneventfully 18 days after surgery, and defecation improved immediately. However, he was readmitted 7 months after surgery with the same abdominal pain and defecation difficulty. A similar finding was found and diagnosed as recurrent volvulus. Therefore, we performed a laparoscopic surgery. The same volvulus as in the previous surgery was confirmed. The site fixed during the previous surgery showed scars, but the afferent limb was free. The dilated ileum that contained the volvulus was excised only on the oral side of the pouch and an intraluminal anastomosis was performed on the anterior wall of the pouch. He had a good postoperative course and was discharged.

**Conclusion:**

Proper diagnosis of volvulus based on the characteristic imaging findings is important. In principle, bilateral row fixation of the rotated ileum is the basic procedure for volvulus. However, fixation with this technique is sometimes difficult. Therefore, this procedure is one of the useful options for the fixation of difficult or recurrent cases.

## Background

Pouch volvulus after proctocolectomy for ulcerative colitis (UC) is a very rare postoperative complication, and the incidence is reported to be 0.18% (3/1700 cases) [1]. Emergency surgery is needed if the intestine is necrotic or if decompression is unsuccessful due to endoscopic reduction. The common site of pouch volvulus has been reported to be the ileal pouch–anal anastomosis and the middle part of the pouch, but no reports on pouch volvulus in the afferent limb of the pouch have been observed [1–9]. In order to preserve pouch and maintain quality of life (QOL), it is necessary to accurately diagnose the site of volvulus and select appropriate treatment strategies. Here, we report the case of a patient with afferent limb volvulus who underwent afferent limbpexy but required reoperation 7 months later. During the second operation, the dilated afferent limb that contained the volvulus was excised only on the oral side of the pouch, and an intraluminal anastomosis was performed on the anterior wall of the pouch using a circular stapler.

## Case presentation

A 38-year-old man with refractory UC had undergone open proctocolectomy (J-pouch–anal hand-sewn anastomosis) 10 years ago at another hospital. He had been aware of lower abdominal pain and bowel movement difficulty for 2 years. He had been admitted to another hospital several times with a diagnosis of bowel obstruction. Although the symptoms were relieved conservatively, there was repeated bowel obstruction caused by volvulus, so he was referred to our hospital for surgery.

**Fig. 1:**
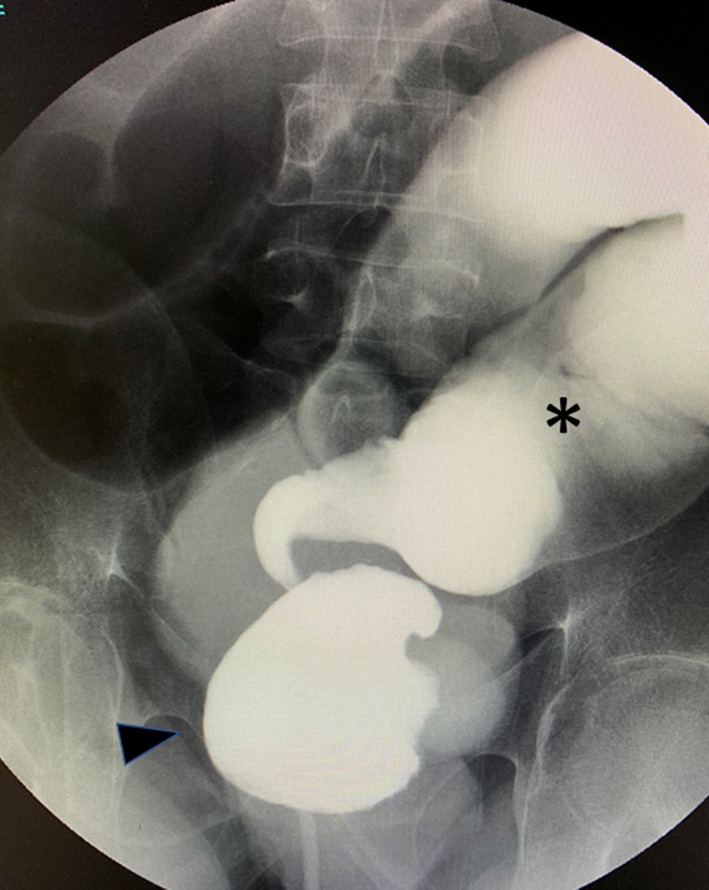
Water-soluble contrast enema demonstrating the corkscrew configuration of the afferent limb of the pouch and dilation of the oral ileum. Black up-pointing triangle: J-pouch, *: dilated oral ileum

Water-soluble contrast enema (Enema) demonstrated a corkscrew configuration of the afferent limb of the pouch and dilation of the oral ileum (Fig. 1). Endoscopically, the lumen was narrowed and twisted at the top of the pouch.

**Fig. 2:**
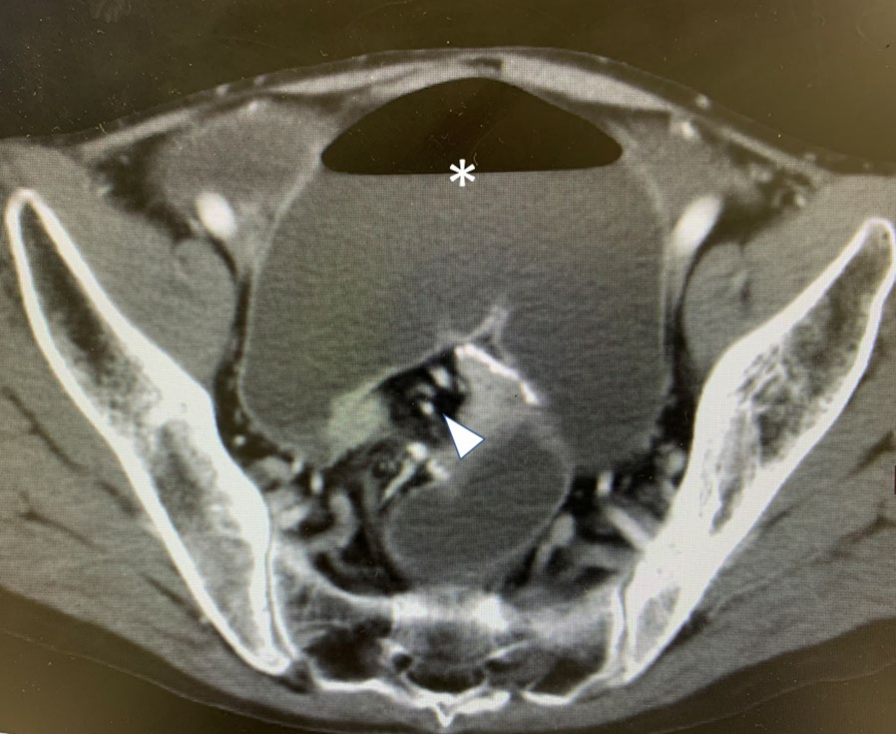
Enhanced multidetector computed tomography demonstrates a whirl sign at the upper corner of the pouch. White up-pointing triangle: superior mesenteric artery and vein, *: dilated oral ileum

Enhanced multidetector computed tomography (MDCT) showed a whirl sign at the upper corner of the pouch and dilatation of the oral ileum, suggesting a volvulus centered on the superior mesenteric artery (SMA) (Fig. 2).

Based on these findings, we made a diagnosis of a pouch volvulus and performed laparoscopic surgery.

## Operation findings and procedure

**Fig. 3:**
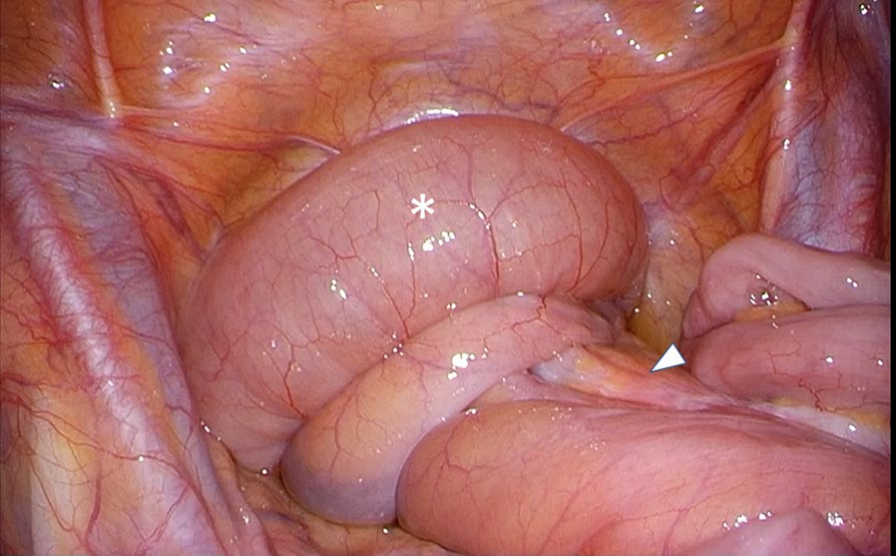
Counterclockwise rotation (270°) of the afferent limb of the pouch was recognized. *: Volvulus in the afferent limb of the pouch, White up-pointing triangle: superior mesenteric artery and vein

**Fig. 4:**
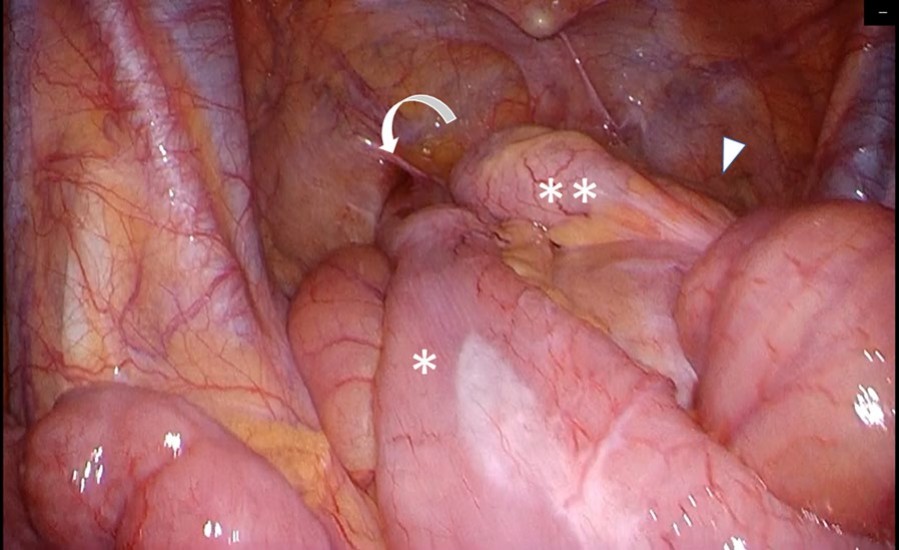
The J-pouch was anastomosed with the trunk with 30-degree clockwise rotation, and there was a space in which the oral ileum hung in an anterior downward direction (white down-pointing triangle). *: Afferent limb, **: efferent limb of the pouch

**Fig. 5:**
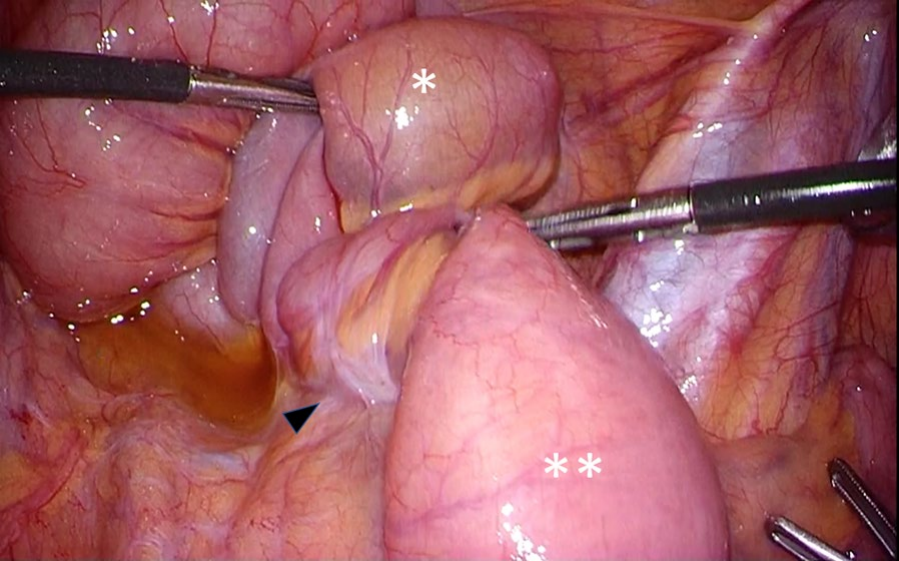
The ileal mesentery was adhered and fixed to the presacrum 20 cm from the oral side of the pouch (black up-pointing triangle). *: Volvulus in the afferent limb, **: oral side of the ileum

**Fig. 6:**
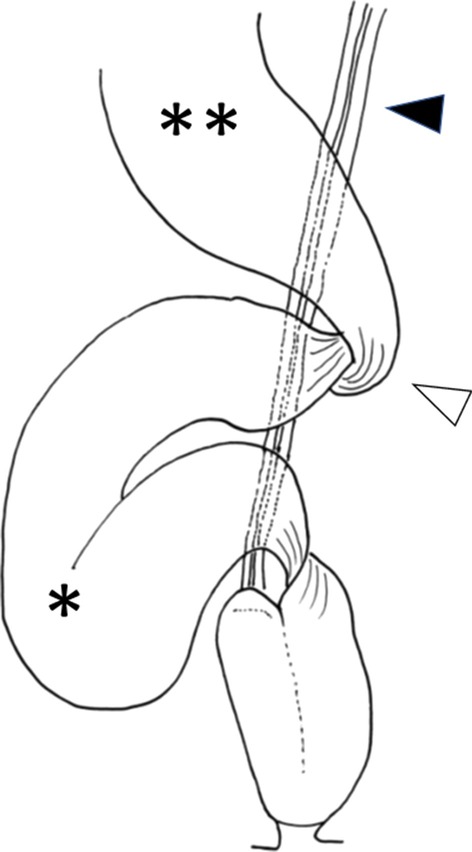
Schematic of the operative findings and procedures showing the volvulus on its long axis. *: Ileal volvulus, **: dilated proximal ileum, black up-pointing triangle: superior mesenteric artery and vein, white up-pointing triangle: adhesion to the presacrum

Laparoscopically, there was a dilated ileum with a volvulus at the bottom of the pelvis. The ileum was dilated around the superior mesenteric artery. Counterclockwise rotation (270°) of the afferent limb of the pouch was recognized (Fig. 3). The J-pouch was anastomosed with the trunk with 30-degree clockwise rotation, and there was a space in which the oral ileum hung in an anterior downward direction (Fig. 4). Moreover, the ileal mesentery was adhered and fixed to the presacral space 20 cm from the oral side of the pouch (Figs. 5, 6). No stricture of the intestinal lumen was observed. Therefore, it was considered that the ileum was rotated between the pouch and the adhesion. No cases of adhesion or internal hernia were found around the pouch.

**Fig. 7:**
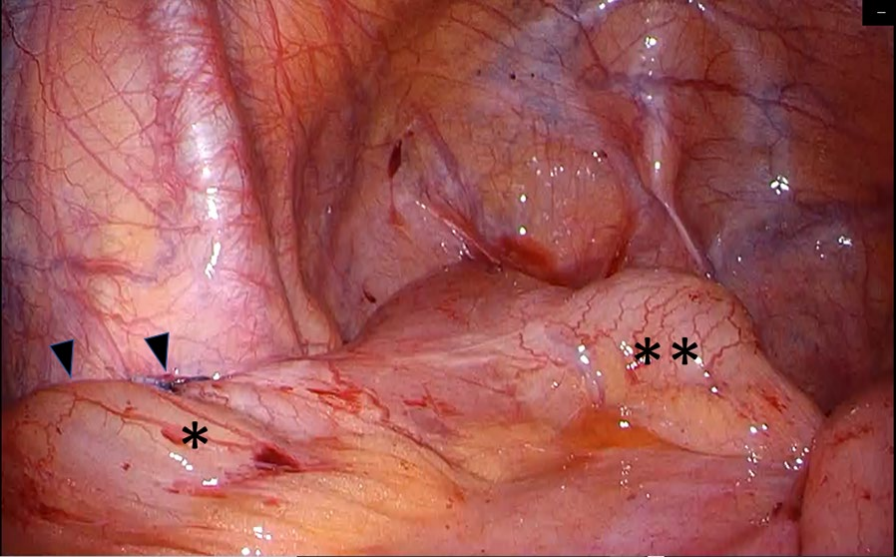
Laparoscopic afferent limbpexy was performed on the left lateral pelvic wall (black up-pointing triangle). *: Afferent limb, **: efferent limb of the pouch

When the afferent limb of the pouch was pulled up, the volvulus was released to a normal state. Therefore, the antimesenteric side of the afferent limb was fixed using three interrupted stiches using 3–0 absorbable braided sutures on the left peritoneal wall of the pelvis (Fig. 7).

His postoperative course was uneventful, and he was discharged 18 days after surgery. Defecation improved immediately after the operation. However, the same abdominal pain and defecation difficulty appeared again 2 months later. He was readmitted 7 months after surgery. A similar finding was found and was diagnosed as a recurrent volvulus. After emergency hospitalization, transanal tubing decompression was performed. Therefore, we performed a laparoscopic surgery.

## Laparoscopic findings

**Fig. 8:**
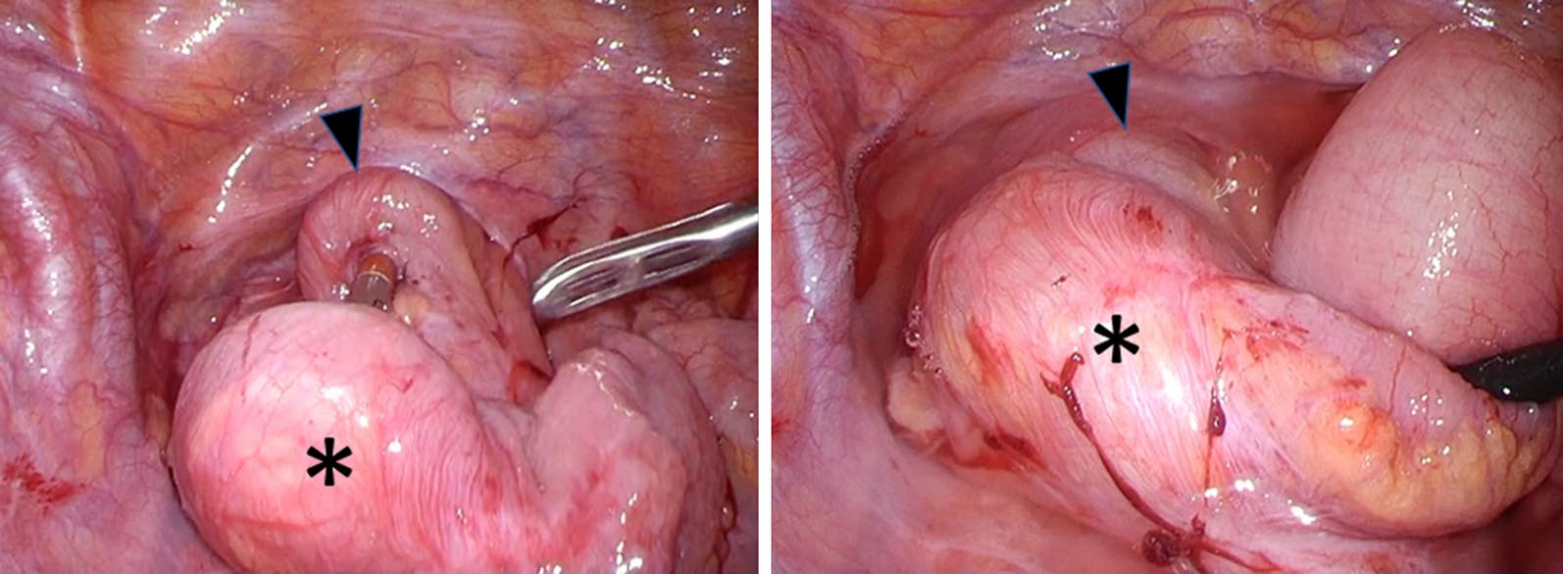
Laparoscopic partial resection of the rotated small and pouch-ileum reanastomosis was performed for recurrent volvulus. *: Oral side of the ileum, black down-pointing triangle: Anterior wall of the pouch

**Fig. 9:**
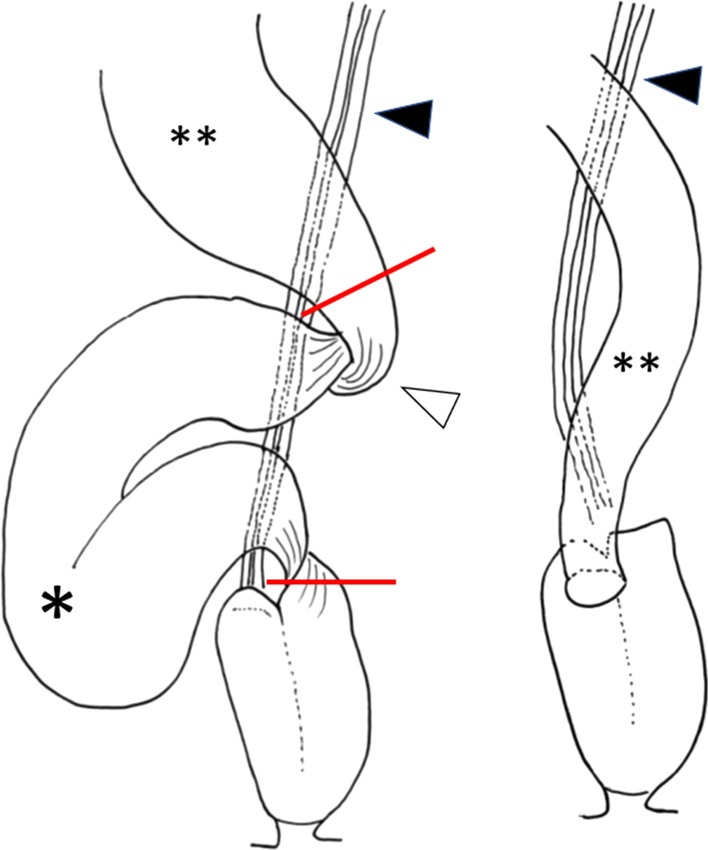
The dilated ileum that contained the volvulus was excised only on the oral side of the pouch, with a length of 18 cm, and an intraluminal anastomosis was performed on the anterior wall of the pouch using a circular stapler. *: ileal volvulus, **: dilated proximal ileum, black up-pointing triangle: superior mesenteric artery and vein, white up-pointing triangle: adhesion to the presacrum

The same volvulus as in the previous operation was confirmed laparoscopically. The site fixed during the previous surgery showed scars on the left pelvic wall, but the afferent limb was free. No adhesions or internal hernias were noted. To avoid damaging the blood flow to the pouch, the dilated ileum that had the volvulus was excised only on the oral side of the pouch with a length of 18 cm, and an intraluminal anastomosis was performed on the anterior wall of the pouch using a circular stapler (Figs. 8, 9).

He had a good postoperative course and was discharged 9 days after the operation.

## Discussion

The common site of a pouch volvulus is reported to be the pouch–anal anastomosis and the middle of the pouch [1–9]. In this case, the afferent limb of the pouch had a long-axis rotated volvulus. Repeated symptoms were also observed in this case, and he was referred to our hospital for the purpose of surgery. Enema and MDCT are useful in the diagnosis of a volvulus, and corkscrew and whirl signs are typical findings [1, 2]. The previous reports showed that the risk factors of pouch volvulus were minimally invasive technique, few adhesions in the pelvis, female sex, low BMI, the oral intestinal tract reaches the pelvis with sufficient margins and an anterior positioning of the pouch mesentery [2, 5]. Few adhesions in the pelvis and the oral intestinal tract reaches the pelvis with sufficient margins were also evident in our case. The difference in our case from the previous report was that the volvulus occurred in the afferent limb of the pouch. In our facility, we perform 90° counterclockwise rotated pouch–anal anastomoses. During this procedure, the SMA is located on the back side of the pouch. In this case, the J-pouch was anastomosed horizontally with the trunk, and there was a space in which the oral ileum hung in an anterior downward direction. Moreover, the ileum was adhered and fixed to the mesentery 20 cm from the oral side of the pouch. Therefore, it was considered that the ileum was rotated between the pouch and the adhesion.

The basic surgical treatment for the prevention of recurrence is fixation of the intestine. In this case, we performed a single-row afferent limbpexy in the afferent limb of the pouch during the first surgery. We used 3–0 absorbable braided sutures for fixation, but it was better to use non-absorbable sutures. Bilateral row fixation of the afferent limb was difficult because the mesenteric arteries and veins were interposed between the limbs and the anterior surface of the sacrum. However, the same symptoms reappeared after 7 months, so we decided to reoperate.

Recurrence after pouchpexy has been reported, and several rows are recommended instead of single rows [3, 4]. Another method has been reported in which the peritoneum is incised and both sides of the pouch are firmly fixed to the anterior surface of the sacrum [5].

During the laparoscopic surgery, the dilated intestine was resected, and the oral intestine was anastomosed to the anterior wall of the ileal pouch using a circular stapler.

It was speculated that there would be a high possibility of recurrence if fixation was performed in the same manner as in the previous surgery. Excising the afferent limb and fixing it to the pelvic wall with mesh around the afferent limb were considered as options. There were two reasons for choosing this procedure. First, it was difficult to fix the afferent limb to the front of the sacrum with mesh, similar to intestinal fusion for rectal prolapse [10]. Second, it was easier to excise the dilated ileum than to perform afferent limb fixation. This procedure is considered to be an application of the usefulness of intestinal resection in sigmoid colon volvulus [11]. The point of caution when performing resection is to ensure blood flow to the pouch. If the blood flow to the pouch is damaged, pouch excision is necessary. Therefore, it is necessary to pay close attention to the preservation of SMA blood flow. The standard surgical procedure for pouch volvulus is pouchpexy to periosteum. The surgical procedure for the volvulus of afferent limb of pouch is different from that for pouch volvulus, and no standard surgical techniques have been proposed.

## Conclusion

Proper diagnosis of volvulus based on the characteristic imaging findings is important. In principle, bilateral row fixation of the rotated ileum is the basic procedure for volvulus. However, this technique is sometimes difficult. Therefore, this procedure is one of the useful options for the fixation of difficult or recurrent cases.

## Data Availability

Not applicable.

## Abbreviations

UC: Ulcerative colitis
Enema: Water-soluble contrast enema
MDCT: Enhanced multidetector computed tomography
SMA: Superior mesenteric artery
